# Dissociation in Effective Treatment and Behavioral Phenotype Between Stress-Enhanced Fear Learning and Learned Helplessness

**DOI:** 10.3389/fnbeh.2019.00104

**Published:** 2019-05-15

**Authors:** Michael A. Conoscenti, Michael S. Fanselow

**Affiliations:** ^1^Department of Psychology, University of California, Los Angeles, Los Angeles, CA, United States; ^2^Staglin Center for Brian and Behavioral Health, University of California, Los Angeles, Los Angeles, CA, United States; ^3^Department of Psychiatry and Biobehavioral Sciences, University of California, Los Angeles, Los Angeles, CA, United States

**Keywords:** learned helplessness, stress-enhanced fear learning, PTSD, depression, fear, stress

## Abstract

Post-traumatic stress disorder (PTSD) is a debilitating disease with relatively high lifetime prevalence. It is marked by a high diversity of symptoms and comorbidity with other psychiatric disease. Furthermore, PTSD has a high level of origin and symptom heterogeneity within the population. These characteristics taken together make it one of the most challenging diseases to effectively model in animals. However, with relatively little headway made in developing effective disease interventions, PTSD remains as a high priority target for animal model study. Learned Helplessness (LH) is a procedure classically used to model depression, but has in recent years transitioned to use as a model of PTSD. Animals in this procedure receive 100 inescapable and unpredictable tailshocks or simple restraint without shock. The following day, the animals are tested in a shuttle box, where inescapably-shocked subjects exhibit exaggerated fear and profound deficit in escape performance. Stress-enhanced fear learning (SEFL) also uses an acute (single session) stressor for modeling PTSD in rodents. The SEFL procedure begins with exposure to 15 footshocks or simple context exposure without shock. Animals that initially received the 15 footshocks exhibit future enhanced fear learning. In this review, we will compare the behavior, physiology, and interventions of these two animal models of PTSD. Despite considerable similarity (a single session containing inescapable and uncontrollable shock) the two procedures produce a very divergent set of behavioral consequences.

Up to 20% of the population that experiences a trauma will go on to develop Post-Traumatic Stress Disorder (Kilpatrick et al., 2013; PTSD). PTSD is a debilitating disease marked by symptoms such as dissociative amnesia, avoidance behaviors, hypervigilance, anhedonia, exaggerated fear startle, and insomnia (Association, 2013). Lifetime prevalence of PTSD in the United States is approximately 7%, with U.S. military incidence reaching as high as 15–20% (Gradus, 2007; Gates et al., 2012). A large ongoing research effort has focused on identifying the neurobiological consequences of stress that lead to the development of disorders such as PTSD. Despite great headway made in understanding the neurobiology of the disease, improvement in efficacious intervention has been bare. This point was highlighted in a recent public message from the Director of the National Institute of Mental Health, Dr. Joshua Gordon. In some cases, this lack of progress has led to criticism of the animal models available to study PTSD.

In a recent review by Richter-Levin, Stork, and Schmidt, the authors weigh-in on the current stress research climate (Richter-Levin et al., 2018). The authors suggest that while animal model research has proved invaluable in the study of PTSD, modifications should be made to adequately capture the complexity and heterogeneity of the disease in order to increase translational relevance. Those authors suggest that animal models of PTSD should be modified to accurately represent exposure to risk factors and individual genetic and behavioral differences. They also suggest careful selection of stressor and behavioral phenotypes measured, suggesting that just as we see in humans, different stressors produce dissociable neurobiological and behavioral consequences in rodents.

In this review, we will examine this notion of stressor-induced heterogeneity. We will critically evaluate the reported effects of two animal stress procedures that have been claimed to model PTSD psychopathology. Learned helplessness (LH) is a half-century old procedure which commonly uses 100, 8 s inescapable and unpredictable 1 mA tailshocks over a 2-h session to produce a behavioral phenotype that parallels many of the symptoms of PTSD and Major Depressive Disorder (MDD). Stress-enhanced fear learning (SEFL) is a procedure that presents 15, 1 s inescapable and unpredictable 1 mA footshocks over a 90-min session to induce its PTSD-like phenotype. By critically examining two models that share several dimensional similarities, we can evaluate the specific consequences of stress volume on stress-induced psychopathology. In this case, we are defining volume as the product of shock number, shock duration, and shock intensity (current). It can also be thought of as the total number of coulombs received during stress. Previous work has shown that variation in coulombs qualitatively changes reactions to a stressor (Fanselow, 1984).

In this review we will focus on learned helplessness and SEFL stress procedures in rats only. SEFL in mice is still in its infancy, and we therefore do not feel comfortable discussing these findings at this time. Furthermore, there are several important changes made to the LH procedure when using mice and there is some controversy due to these changes (Landgraf et al., 2015).

## Learned Helplessness

### History

The learned helplessness procedure is a traditional method for analyzing the effects of acute, traumatic stress and modeling related symptoms of post-traumatic stress disorder and comorbid major depression in rats (Minor et al., 1991, 2010; Başoglu et al., 1997; Hammack et al., 2012; Minor and Plumb, 2012). Seligman and colleagues first discovered in 1967 that exposure to inescapable shock, but not escapable shock, results in failure to perform future escape responding in a novel apparatus (Overmier and Seligman, 1967; Seligman and Maier, 1967). The classic experiments utilized dogs and a triadic design. In this design there are three groups. One group is able to perform a response to escape the shock. Another group is able to perform the same response non-contingently, as their exposure to shock is yoked to that of the escapable group. A final group is exposed to the same apparatus, but no shock is administered. This design allows for dissociable assessment of the effects of escapable and inescapable shock. The term “learned helplessness” was originally coined as it was initially believed that the escape latency deficits were due to the animals learning that they had no control over the environment (Seligman and Maier, 1967; Maier and Seligman, 2016). However, others have provided subsequent evidence which has suggested that it instead may be the unpredictability of shock that is the root of the subsequent maladaptive behavior (Dess et al., 1990; Minor et al., 1991; Minor and Hunter, 2002). The model has since transitioned to rats and LH has been used extensively as an animal model of human disorders, such as PTSD and MDD (Maier, 1984; Foa et al., 1992). Though the learned helplessness model has been used extensively as a model of depression and PTSD, it does have a scientifically contentious history. The relatively short 24–72-h lifespan of many of the observed behavioral and cognitive deficits, which can be moderately extended using a reinstatement procedure (Maier, 2001), has been a point of which its opponents cite when discussing its inefficacy as a model of psychiatric disease (Anisman and Sklar, 1979; Jackson et al., 1979; Minor et al., 1988; Dess et al., 1989; Yehuda and Antelman, 1993). However, face, construct, and predictive validity maintain its place as one of the leading models of PTSD and MDD.

It should be noted that it is common to drop the escapable group in studies more concerned with modeling human stress disorders, and less concerned with questions on the effects of escapability. Both SEFL and the LH procedures induce behavioral changes by delivering inescapable shocks but the two procedures differ substantially in terms of the amount of shock delivered. Seeing as the effect of shock volume, and not escapability, is the focus of this review, we will be discussing the overall behavioral and physiological consequences of inescapable shock and not learned helplessness, *per se*. In other words, we will not disentangle if the effects discussed are specific to inescapable shock or if they also occur in rats that receive equivalent escapable shock (see Greenwood and Fleshner, 2008; Maier and Seligman, 2016 for review on the behavioral effects of escapability).

### Induced Phenotype

Animals exposed to 100 inescapable and unpredictable shocks exhibit several behavioral characteristics similar to the symptoms of PTSD (see Table 1). Rats pre-exposed to inescapable shock enter the subsequent test situation in an anxious/agitated state and show exaggerated fear responding during initial escape testing. As testing progresses, inescapably shocked rats rapidly transition to an unresponsive, depression-like state, termed *conservation-withdrawal*. The transition to conservation-withdrawal is evident as a profound deficit in escape performance (Minor et al., 1994a,b; Plumb et al., 2013). Experience with inescapable shock also results in behavioral depression as defined by the forced swim task (Weiss et al., 1981) and sucrose preference (Christianson et al., 2008; but see Dess, 1992), disturbances in sleep (Adrien et al., 1991), exaggerated startle (Servatius et al., 1995), anorexia (Weiss, 1968; Dess et al., 1989), anhedonia (Zacharko and Anisman, 1991), anxiety as measured by decreased social interaction (Short and Maier, 1993) and the elevated plus maze (Steenbergen et al., 1989), reinstatement of drug seeking (Figueroa-Guzman et al., 2011) and attentional/cognitive deficits in rats (Jackson et al., 1980; Minor et al., 1984; Shors, 2004). However, it should be noted that many of the behavioral deficits are short lived and fail to occur 72 or more hours following the traumatic stress session (Jackson et al., 1978; Grau et al., 1981; Weiss et al., 1981; Maier, 1990; Short and Maier, 1993; Will et al., 1998). Several of the neurochemical changes induced by inescapable shock also persist for only a few days (Weiss et al., 1981; Maier, 2001).

**Table 1:** Summary of LH and SEFL-induced change.

**Phenotype**	**Present in LH?**	**Present in SEFL?**	**References**
Future enhanced fear learning	Yes	Yes	(Rau et al., 2005; Baratta et al., 2007; Rau and Fanselow, 2009)
Anxiety; Elevated plus maze	Yes	Yes	(Steenbergen et al., 1989; Poulos et al., 2014)
Anxiety; Open field	Yes	Yes	(Fleshner and Greenwood, 2013; Perusini et al., 2016)
Anxiety; Exaggerated startle	Yes	Yes	(Servatius et al., 1995; Perusini et al., 2016)
Anxiety; Social interaction	Yes	Not reported	(Short and Maier, 1993)
Depression; Shuttle escape deficit	Yes	No	(Seligman and Maier, 1967; Minor et al., 1994a)
Depression; Forced swim	Yes	Maybe	(Weiss et al., 1981; Perusini et al., 2016; Tribble and Fanselow, 2019)
Depression; Sucrose preference	Yes	Not reported	(Dess, 1992; Christianson et al., 2008)
Anorexia	Yes	Not reported	(Weiss, 1968; Dess et al., 1989)
Reinstatement of drug seeking	Yes	Yes	(Figueroa-Guzman et al., 2011; Meyer et al., 2013)
**NEUROBIOLOGY**			
Amygdala	Yes	Yes	(Maier et al., 1993; Perusini et al., 2016)
Ventromedial prefrontal cortex	Yes	Yes	(Maier and Seligman, 2016; Pennington et al., 2017)
Dorsal raphe nuclei	Yes	Not reported	(Maier and Seligman, 2016)
Nucleus accumbens	Yes	Not reported	(Plumb et al., 2013)
Dorsal striatum	Yes	Not reported	(Strong et al., 2011)
BNST	Yes	Not reported	(Hammack et al., 2004, 2012)
Habenula	Yes	Not reported	(Dolzani et al., 2016)
Corticosterone	Yes	Yes	(Hanff et al., 2010; Poulos et al., 2014; Perusini et al., 2016)
Serotonin	Yes	Not reported	(Maier and Seligman, 2016)
Norepinephrine	Yes	Not reported	(Minor et al., 1988; Grahn et al., 2002)
Interleukin-1	Yes	Yes	(Goshen and Yirmiya, 2009; Jones et al., 2015)
Glucose	Yes	Not reported	(Minor and Saade, 1997; Conoscenti et al., 2017)
Adenosine	Yes	Not reported	(Minor et al., 1994a,b; Plumb et al., 2013)

*This table displays a summary of the behavioral, neural, and pharmacological effects of LH and SEFL stressors*.

Interestingly, this severe stress procedure does not appear to enhance future fear learning as appreciably as the more moderate, SEFL stress procedure. One notable study provides evidence that inescapable shock may enhance, while escapable shock reduce, subsequent fear learning (Baratta et al., 2007). Furthermore, in a similar stress protocol (of considerably smaller total stress volume), inescapable tailshock has also been shown to enhance trace eyeblink conditioning (Beylin and Shors, 1998) However, it should be noted that the effects observed in both are relatively modest in comparison to the effect found using the moderate, SEFL stress procedure.

### Behavioral Interventions

Several behavioral factors and interventions have profound effects on the phenotype produced by severe stress. For example, rats no longer exhibit post-stress escape latency deficits if they are given 6 weeks of free access to a running wheel prior to the trauma, and the protective effects of wheel running are dependent on the duration of activity (Greenwood et al., 2005). Perhaps more surprisingly, prior exposure to subthreshold stress has also exhibited beneficial effects following exposure to the traumatic stress session (Plumb et al., 2015).

Several design aspects are critical in the development of the phenotype produced by severe stress. For one, though the pretreatment and testing contexts differ on many dimensions, shuttle-escape deficits are contingent upon the stress and test contexts sharing the same olfactory cues (Minor and LoLordo, 1984). Traditionally, this is done by allowing feces and urine of the stressed animals to accumulate over the day. If one of the contexts is cleaned, the learned helplessness phenotype is abolished. Furthermore, if the contexts are cleaned and instead both scented with a common artificial odor, the behavioral phenotype persists. It should be noted that the effect of contextual odor generalization has not been tested for other behaviors induced by inescapable shock and does not likely play a similar role. Another essential dimension of the design is that the shocks remain variable and unsignaled. If the shocks are cued, the behavioral phenotype no longer persists (Dess et al., 1990).

### Pharmacological Interventions/Defined Neurocircuitry

Research into the neural mechanisms of the behavioral consequences of severe stress was spearheaded early on by Steve Maier. Through decades of research, the Maier lab has characterized the importance of serotonin (5-HT) signaling in the dorsal raphe nucleus (DRN) in the development of the LH phenotype (for review, see Maier and Seligman, 2016). Within this model, he proposes that DRN activity is modulated by the controllability of the stressor via detection and activation in the ventromedial prefrontal cortex (vmPFC). For example, activation of the vmPFC using picrotoxin eliminates subsequent LH behavior in rats exposed to inescapable shock (Amat et al., 2008). Through Maier's body of work, he also implicates roles of the bed nucleus of the stria terminalis (Hammack et al., 2004, 2012), amygdala (Maier et al., 1993), and dorsal striatum (Strong et al., 2011). The habenula- DRN circuit has also been identified to play a role using a unique behavioral outcome in juvenile rats (Dolzani et al., 2016; see Metzger et al., 2017 for review). Additionally, research from several labs has suggested an integral role of norepinephrine signaling in the development on LH behaviors (Minor et al., 1988; Grahn et al., 2002).

Thomas Minor focused on the energetic demands of the stressor as a critical aspect that leads to future maladaptive behavior in the animal. Minor suggests both serotonin and corticosterone likely play only permissive roles in the development of the behavioral consequences induced by severe stress. This is based on their time course of release during stress exposure and testing (see Minor and Hunter, 2002 for review). Instead, he suggests that the state of fear invoked by the stress session is energetically costly and depletes the animal's energy reserves (Conoscenti et al., 2019). Thus, the animal enters the test session in a state of conservation withdrawal, a behavior deemed to conserve energy resources. This behavior limits the animal's motivation to escape, and is mediated by adenosine signaling in the nucleus accumbens core (Minor et al., 1994a,b, 2001, 2006, 2008, 2010; Plumb et al., 2013). Furthermore, consumption of glucose following the trauma, which has been shown to replete energy reserves (Conoscenti et al., 2019), eliminates the negative behavioral consequences of stress (Minor and Saade, 1997; Conoscenti et al., 2017, 2019). This theory accounts for the transient nature of the behavioral effects, as the effects disappear as the animal recovers from the energy deficit. However, it should be noted that it does not account for experiments showing that inescapably shocked rats with amygdalar lesions will still exhibit shuttle escape latencies despite lacking a fear response (Maier et al., 1993).

Another line of evidence implicates the role of the immune response in the development of LH behavior. Specifically, several studies have suggested that interleukin-1 (IL-1), an inflammatory cytokine, is critical for LH's characteristic shuttle escape latency deficits (Maier and Watkins, 1995; Minor et al., 2006; Goshen and Yirmiya, 2009; Hanff et al., 2010). Following inescapable shock, hippocampal, hypothalamic, and peripheral concentrations of IL-1 increase. This upregulation of IL-1 is necessary, but not sufficient, for the induction of stress-induced behavior, as it has been shown that blocking IL-1 mitigates the behavioral consequences of shock. It has been posited that IL-1 exerts its stress mediating effects by inducing an increase in HPA-axis activation (see Goshen and Yirmiya, 2009 for review).

## Sefl

### History

**Figure 1:**
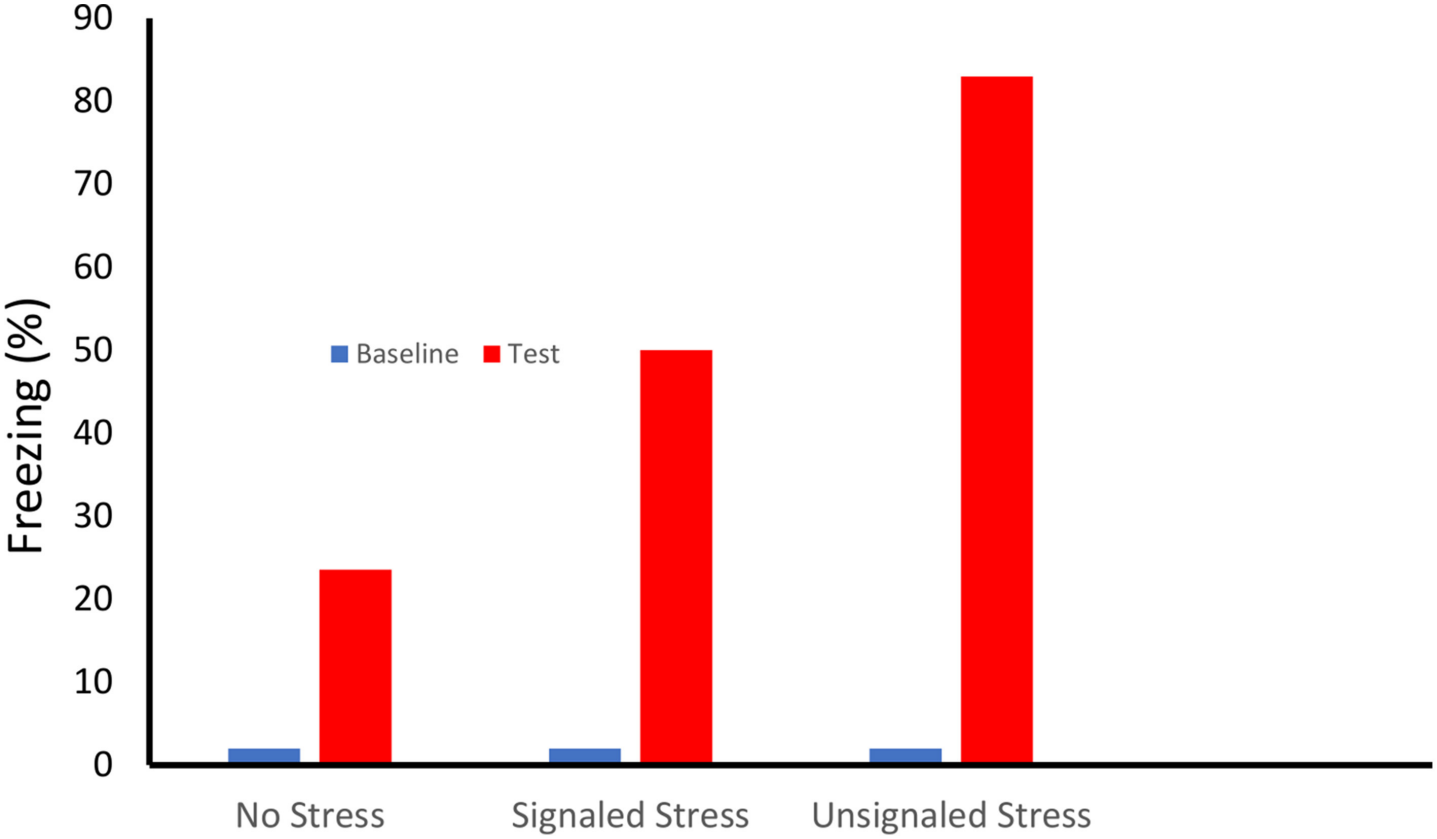
On Day One female Long-Evans rats received either no treatment, or 15 shocks (0.71-mA, 0.75-s) that were either preceded by a 30 s tone (Signaled Stress) or followed by the tone (Unsignaled Stress) in a rectangular shuttle box. Subsequently the rats received a single conditioning shock (1.0-mA, 0.75-s) in a conditioning chamber that differed in term of shape, smell, location, dimensions and lighting. Prior to the conditioning shock the there was little freezing (< 2%) in the conditioning chamber. Animals that received a prior signaled shock stressor showed more than twice the level of freezing of the unstressed controls. Fear learning showed an even greater enhancement in that rats whose stress was unsignaled [Based on Fanselow and Bolles (1979a,b)].

Our first indication of enhanced fear learning following stress was suggested by two papers published in 1979 (Fanselow and Bolles, 1979a,b). In these experiments rats that received an identical single shock in the same novel context froze at very different rates depending on whether or not they received prior experience with a robust fear conditioning protocol in a completely different context (see Figure 1). Interestingly, while both 15 forward (tone-shock) and backward (shock-tone) trials enhanced subsequent contextual fear conditioning, predictive signaling of the shock reduced the magnitude of this enhancement. Importantly, the lack of freezing observed prior to the single shock indicated that this enhancement was not caused by generalization of fear from the 15 shock to the 1 shock contexts.

This ability of stress to enhance fear learning was then used as a tool to explore two deficits in contextual fear conditioning (Fanselow et al., 1993). One was the deficit seen when only a minimal period of exploration was allowed prior to delivery of a single shock. Prior stress facilitated conditioning with this procedure that typically supports little to no conditioning. Another deficit in contextual fear conditioning occurs when shocks are closely spaced rather than given in a more distributed manner. In this case, prior stress eliminated the difference between massed and spaced trials. These studies also revealed an important boundary conditioning to SEFL; when multiple conditioning shocks were well spaced prior stress caused no enhancement in fear learning. These findings indicate that stress enhances the rate but not the asymptote of the learning curve.

### Induced Phenotype

As previously discussed, the quintessential phenotype measured using this model is the enhancement of future fear learning (Rau et al., 2005; Rau and Fanselow, 2009). However, animals exposed to 15 inescapable and unpredictable shocks also exhibit several similar symptoms to LH-stressed animals (see Table 1). Animals exposed to 15 footshocks exhibit reinstatement of drug seeking (Meyer et al., 2013) as well as several anxiety-like phenotypes. For example, animals that receive shock exhibit decreased time in the open arms of the elevated plus maze (Poulos et al., 2014), decreased exploration during an open field test (Perusini et al., 2016), and potentiated startle (Perusini et al., 2016). Unlike LH-stressed animals, there is no evidence suggesting that these animals exhibit depression-like behavior after to exposure to 15 shocks. With this stress volume, animals fail to exhibit escape latency deficits (Minor et al., 1994c), though it should be noted that this study used tailshock, not footshock. While one study did show an effect of shock on float time in the forced swim test (Perusini et al., 2016), this effect has subsequently failed to replicate (Tribble and Fanselow, 2019). Interestingly, the behavioral effects of the SEFL stress have been shown to persist for several months (Rau and Fanselow, 2009). This symptom persistence is notable, seeing that the LH procedure has a much higher stress volume and yet several of the behavioral effects are much more transient in nature.

Unlike shuttle-escape performance deficits in LH, it appears that the SEFL behavior does not rely on associative processes such as context generalization (Rau et al., 2005; Poulos et al., 2014; Pennington et al., 2017). Stress during adolescence still results in SEFL even though this early life stress does not produce associative fear learning (Poulos et al., 2014). SEFL behavior is also resistant to extinction of the trauma context, further suggestion that there are non-associative processes at play (Rau et al., 2005; Long and Fanselow, 2012). However, it has been hypothesized that perhaps animals that undergo the SEFL procedure are learning a shock-shock association. That is, the animal is learning that one footshock predicts subsequent footshock, and the enhancement of fear to the 1-shock context is due to this learned association. To support the notion that the effects of SEFL are not due to a shock-shock association, we have found that stress pretreatment exposure will enhance subsequent fear learning when using a loud noise as the stressor (Pennington et al., 2017). Furthermore, the SEFL effects no longer appear if the 1-shock exposure precedes the 15-shock session (Rau et al., 2005). It should be noted that while this evidence does not eliminate the possibility of shock-shock associations from playing a role in SEFL, this explanation is less applicable to the behavioral changes produced by LH-stress due to their transituational nature.

### Behavioral Interventions

The SEFL phenotype is relatively robust, and therefore has seen little success in terms of behavioral interventions. In the majority of stress models, animals are singly housed, as pair-housed animals often show decreased behavioral effects of stress (Liu et al., 2013). However, a series of studies aimed at probing the effects of single vs. pair-housing animals showed no significant effects in eliminating the SEFL phenotype (Tribble and Fanselow, 2019).

Several aspects of SEFL design are in direct contrast with the LH stress procedure. The most apparent is that the SEFL behavior is not dependent on shared cues between contexts. Indeed, great care is taken in the SEFL procedure to eliminate any similarity between the stress and conditioning contexts. Additionally, the effects of signaling shock show slightly different outcomes. As previously mentioned, evidence suggests that signaling shock during stress pre-exposure may act to reduce, but not eliminate, SEFL behavior (Fanselow and Bolles, 1979a,b).

### Pharmacological Interventions/Defined Neurocircuitry

Compared to the decades of research dedicated to identifying the neural mechanisms of LH behavior, the neurocircuitry of SEFL behavior remains relatively scant (see Table 1). Similar to LH, it appears that corticosterone is necessary, but not sufficient, for the induction of SEFL behavior (Perusini et al., 2016). Furthermore, the SEFL stress procedure produces a similar dysregulation of the diurnal cycle of corticosterone (Poulos et al., 2014). Finally, a series of studies suggest that glucocorticoids may be acting via activation glucocorticoid receptors in the basolateral amygdala, which in turn upregulate the GluA1 AMPA receptor subunit in this structure (Perusini et al., 2016).

It appears that the ventromedial prefrontal cortex also plays an important role in the SEFL phenotype. A study showed that when the vmPFC is lesioned, future enhanced fear learning is attenuated, while the trauma memory remains intact (Pennington et al., 2017). Interestingly, this means that the impacts of the vmPFC on LH and SEFL are opposite: activation of vmPFC may be necessary for SEFL, while inactivation of vmPFC during stress pretreatment appears to be necessary for the formation of LH behaviors (Amat et al., 2005, 2008).

Stress-induced immune reactivity also appears to play an essential role in SEFL. Donald Lysle has reported a series of studies which suggest that IL-1beta, specifically, is necessary for the induction of the SEFL phenotype (Jones et al., 2015, 2018). Similar to LH, IL-1 antagonists block the induction of SEFL and shock stress increases both central and peripheral concentrations of IL-1. Furthermore, repeated morphine injection into the dorsal hippocampus following stress pretreatment has been reported to eliminate the stress-induced increases in IL-1 and subsequent SEFL behavior (Szczytkowski-Thomson et al., 2013; Jones et al., 2015, 2018).

### Summary and Conclusions

In this review, we discussed the behavioral and physiological consequences of two acute stress paradigms that vary on one major dimension: volume. Exposure to inescapable, unpredictable shock appears to incorporate some homogenous peripheral and central mechanisms and induce a series of consistent trans-situational behaviors, regardless of volume. It appears that stress-induced anxiety phenotypes are first to arise during exposure to a stressor, as anxiety-related behaviors are conserved across the two shock-stress models. The HPA axis appears to play a critical, permissive role in the development of both LH and SEFL-induced behavior. It also appears that the immune response, specifically IL-1, plays a critical role in the development of stress-induced psychopathology. Regarding neurocircuitry, converging evidence suggests that the amygdalar complex is involved in the neurocircuitry of shock stress regardless of volume. The vmPFC has also been implicated in both behavioral models, though it appears to have opposing effects.

Several dissociable behavioral and neurobiological aspects of the two procedures stand out. The most obvious division is the induction of a depression-like phenotype in LH-stressed animals that appears absent in SEFL-stressed animals. Another interesting difference is the apparent generalization necessary for LH's characteristic deficits in shuttle-escape performance, which does not appear necessary for the SEFL phenotype. Perhaps the most perplexing difference is that of symptom persistence. The LH-stressor produces many behavioral changes that appear to persist for only a few days. Meanwhile, SEFL produces a set of behaviors which persist for at least several months. Given that there is a much greater volume of stress in the LH procedure it is surprising that many of its effects do not persevere. However, it should be noted that several of these short-lived changes are in behaviors that do not overlap with the behavioral effects of SEFL. Therefore, it may be a product of the behavioral phenotype assayed, and not an effect directly related to stress volume. It is important to note that there are several outstanding questions that have been left unanswered. For example, the role of 5-HT neurons in the DRN has been well characterized in LH, but has yet to be investigated in SEFL.

Use of the same stressor can produce dissociable behavioral and neural consequences by simply modulating stress volume. Notably, the degree of stress does not necessarily make the effects quantitatively greater, but rather there seems to be qualitative changes in the consequent behavioral reactions. Based on the literature reviewed, it appears that the SEFL procedure may produce several phenotypes specific to model PTSD without depression comorbidity, while LH may model a PTSD comorbid with depression. This notion sits perfectly in-line with the heterogeneity of PTSD described in the review by Richter-Levin et al. (2018). Within that review, the authors describe an outstanding fundamental question about PTSD: is PTSD with depression a unique subtype, or do the diseases merely show a high comorbidity. Approximately half of patients diagnosed with PTSD also concurrently meet criteria for Major Depressive Disorder (Kessler et al., 1995; Breslau et al., 1997; Rytwinski et al., 2013; Caramanica et al., 2014; Flory and Yehuda, 2015). Perhaps even more staggering is the statistic that 95% of those with PTSD will be diagnosed with MDD within their lifetime (Hammack et al., 2012). Patients with MDD exhibit symptoms such as chronic depressed mood, anhedonia, anorexia or hyperphagia, insomnia or hypersomnia, fatigue, and cognitive deficits (Association, 2013). These symptoms are consistent with several of the symptoms observed following LH, but not SEFL, stress exposure. It is possible that human PTSD development is influenced by similar factors. For example, stress volume may influence both the quality and quantity of symptoms. It is also possible, that disease persistence does not positively correlate with stress volume, but may be predicted by another variable of stress exposure. Only through careful, focused study examining the neurobiological effects of modulating stress volume may we begin to unravel the dissociable aspects of PTSD and PTSD with comorbid depression.

Further precise exploration to assess the behavioral and neurobiological dissociation between the two procedures is necessary. By further understanding the mechanisms of each stressor we may be able to more accurately target investigation into neural mechanisms and effective treatment of specific disease phenotypes. This goal can best be reached by minimizing the lab-specific stress procedure permutations that are presently under use and focusing on stressors that can be parametrically titrated and objectively compared.

## Article Dedication

We dedicate this article to the memory of Dr. Thomas R. Minor, a major contributor to the reviewed work on learned helplessness. Tom was an exceptional mentor, a supportive colleague, and a caring friend whose ideas both challenged and sparked our scientific endeavors.

## Author Contributions

All authors listed have made a substantial, direct and intellectual contribution to the work, and approved it for publication.

### Conflict of Interest Statement

The authors declare that the research was conducted in the absence of any commercial or financial relationships that could be construed as a potential conflict of interest.

## References

[B1] Adrien J. Dugovic C. Martin P. (1991). Sleep-wakefulness patterns in the helpless rat. Physiol. Behav. 49, 257–262. 10.1016/0031-9384(91)90041-L 2062895

[B2] AmatJ.BarattaM. V.PaulE.BlandS. T.WatkinsL. R.MaierS. F. (2005). Medial prefrontal cortex determines how stressor controllability affects behavior and dorsal raphe nucleus. Nat. Neurosci. 8, 365. 10.1038/nn139915696163

[B3] Amat J. Paul E. Watkins L. Maier S. (2008). Activation of the ventral medial prefrontal cortex during an uncontrollable stressor reproduces both the immediate and long-term protective effects of behavioral control. Neuroscience 154, 1178–1186. 10.1016/j.neuroscience.2008.04.005 18515010 PMC2862730

[B4] Anisman H. Sklar L. S. (1979). Catecholamine depletion in mice upon reexposure to stress: mediation of the escape deficits produced by inescapable shock. J. Comp. Physiol. Psychol. 93, 610. 10.1037/h0077603 573289

[B5] Association A. P. (2013). Diagnostic and statistical manual of mental disorders (DSM-5®). Washington, DC: American Psychiatric Pub.

[B6] Baratta M. Christianson J. Gomez D. Zarza C. Amat J. Masini C. (2007). Controllable versus uncontrollable stressors bi-directionally modulate conditioned but not innate fear. Neuroscience 146, 1495–1503. 10.1016/j.neuroscience.2007.03.042 17478046 PMC1978104

[B7] Başoglu M. Mineka S. Paker M. Aker T. Livanou M. Gök S. (1997). Psychological preparedness for trauma as a protective factor in survivors of torture. Psychol. Med. 27, 1421–1433. 10.1017/S0033291797005679 9403913

[B8] Beylin A. V. Shors T. J. (1998). Stress enhances excitatory trace eyeblink conditioning and opposes acquisition of inhibitory conditioning. Behav. Neurosci. 112, 1327. 10.1037/0735-7044.112.6.1327 9926816

[B9] Breslau N. Davis G. C. Peterson E. L. Schultz L. (1997). Psychiatric sequelae of posttraumatic stress disorder in women. Arch. Gen. Psychiatry 54, 81–87. 10.1001/archpsyc.1997.01830130087016 9006404

[B10] Caramanica K. Brackbill R. M. Liao T. Stellman S. D. (2014). Comorbidity of 9/11-Related PTSD and depression in the world trade center health registry 10–11 years postdisaster. J. Traumatic. Stress 27, 680–688. 10.1002/jts.21972 25470556

[B11] Christianson J. P. Paul E. D. Irani M. Thompson B. M. Kubala K. H. Yirmiya R.. (2008). The role of prior stressor controllability and the dorsal raphe nucleus in sucrose preference and social exploration. Behav. Brain Res. 193, 87–93. 10.1016/j.bbr.2008.04.024 18554730 PMC2583404

[B12] Conoscenti M. Hart E. Smith N. Minor T. (2017). Temporal parameters of post-stress prophylactic glucose treatment in rats. Stress 20, 1–37. 10.1080/10253890.2017.1334052 28532277

[B13] Conoscenti M. A. Williams N. M. Turcotte L. P. Minor T. R. Fanselow M. S. (2019). Post-stress fructose and glucose ingestion exhibit dissociable behavioral and physiological effects. Nutrients 11:361. 10.3390/nu11020361 30744115 PMC6412320

[B14] Dess N. K. (1992). Divergent responses to saccharin vs. sucrose availability after stress in rats. Physiol Behav. 52, 115–125. 10.1016/0031-9384(92)90440-D 1528993

[B15] Dess N. K. Minor T. Trauner M. Lee C. (1990). Modeling the signal features of an escape response: the effects of cessation conditioning in the“ learned helplessness” paradigm. J. Exp. Psychol. 16, 123–136. 10.1037//0097-7403.16.2.123 2335768

[B16] Dess N. K. Minor T. R. Brewer J. (1989). Suppression of feeding and body weight by inescapable shock: Modulation by quinine adulteration, stress reinstatement, and controllability. Physiol. Behav. 45, 975–983. 10.1016/0031-9384(89)90224-2 2780883

[B17] Dolzani S. D. Baratta M. V. Amat J. Agster K. L. Saddoris M. P. Watkins L. R.. (2016). Activation of a habenulo–raphe circuit is critical for the behavioral and neurochemical consequences of uncontrollable stress in the male rat. eNeuro. 3:ENEURO.0229-16.2016. 10.1523/ENEURO.0229-16.2016 27785462 PMC5066263

[B18] Fanselow M. DeCola J. P. Young S. L. (1993). Mechanisms responsible for reduced contextual conditioning with massed unsignaled unconditional stimuli. J. Exp. Psychol. 19, 121. 10.1037/0097-7403.19.2.121 8505593

[B19] Fanselow M. S. (1984). Shock-induced analgesia on the formalin test: effects of shock severity, naloxone, hypophysectomy, and associative variables. Behav. Neurosci. 98:79. 10.1037//0735-7044.98.1.79 6320845

[B20] Fanselow M. S. Bolles R. C. (1979a). Triggering of the endorphin analgesic reaction by a cue previously associated with shock: reversal by naloxone. Bull. Psychon. Soc. 14, 88–90. 10.3758/BF03329408

[B21] Fanselow M. S. Bolles R. C. (1979b). Naloxone and shock-elicited freezing in the rat. J. Comp. Physiol. Psychol. 93, 736. 10.1037/h0077609 479405

[B22] Figueroa-Guzman Y. Mueller C. Vranjkovic O. Wisniewski S. Yang Z. Li S.-J. (2011). Oral administration of levo-tetrahydropalmatine attenuates reinstatement of extinguished cocaine seeking by cocaine, stress or drug-associated cues in rats. Drug Alcohol Depend. 116, 72–79. 10.1016/j.drugalcdep.2010.11.023 21196089 PMC3466100

[B23] Fleshner M. Greenwood B. N. (2013). Mechanisms Underlying the Relationship Between Physical Activity and Anxiety: Animal Data, Routledge Handbook of Physical Activity and Mental Health. (London: Routledge), 152–64.

[B24] Flory J. D. Yehuda R. (2015). Comorbidity between post-traumatic stress disorder and major depressive disorder: alternative explanations and treatment considerations. Dialogues Clin. Neurosci. 17, 141. 26246789 10.31887/DCNS.2015.17.2/jflory PMC4518698

[B25] Foa E. B. Zinbarg R. Rothbaum B. O. (1992). Uncontrollability and unpredictability in post-traumatic stress disorder: an animal model. Psychol. Bull. 112, 218. 10.1037/0033-2909.112.2.218 1454893

[B26] Gates M. A. Holowka D. W. Vasterling J. J. Keane T. M. Marx B. P. Rosen R. C. (2012). Posttraumatic stress disorder in veterans and military personnel: epidemiology, screening, and case recognition. Psychol. Serv. 9, 361–382. 10.1037/a0027649 23148803

[B27] Goshen I. Yirmiya R. (2009). Interleukin-1 (IL-1): a central regulator of stress responses. Front. Neuroendocrinol. 30, 30–45. 10.1016/j.yfrne.2008.10.001 19017533

[B28] Gradus J. L. (2007). Epidemiology of PTSD. National Center for PTSD. Washington, DC: United States Department of Veterans Affairs.

[B29] Grahn R. E. Hammack S. Will M. O'Connor K. Deak T. Sparks P.. (2002). Blockade of alpha1 adrenoreceptors in the dorsal raphe nucleus prevents enhanced conditioned fear and impaired escape performance following uncontrollable stressor exposure in rats. Behav. Brain Res. 134, 387–392. 10.1016/S0166-4328(02)00061-X 12191825

[B30] Grau J. W. Hyson R. L. Maier S. F. Madden J. Barchas J. D. (1981). Long-term stress-induced analgesia and activation of the opiate system. Science 213, 1409–1411. 10.1126/science.7268445 7268445

[B31] Greenwood B. N. Fleshner M. (2008). Exercise, learned helplessness, and the stress-resistant brain. Neuromol Med. 10, 81–98. 10.1007/s12017-008-8029-y 18300002

[B32] Greenwood B. N. Foley T. E. Burhans D. Maier S. F. Fleshner M. (2005). The consequences of uncontrollable stress are sensitive to duration of prior wheel running. Brain Res. 1033, 164–178. 10.1016/j.brainres.2004.11.037 15694921

[B33] Hammack S. E. Cooper M. A. Lezak K. R. (2012). Overlapping neurobiology of learned helplessness and conditioned defeat: implications for PTSD and mood disorders. Neuropharmacology 62, 565–575. 10.1016/j.neuropharm.2011.02.024 21396383 PMC3433056

[B34] Hammack S. E. Richey K. J. Watkins L. R. Maier S. F. (2004). Chemical lesion of the bed nucleus of the stria terminalis blocks the behavioral consequences of uncontrollable stress. Behav. Neurosci. 118:443. 10.1037/0735-7044.118.2.443 15113272

[B35] Hanff T. C. Furst S. J. Minor T. R. (2010). Biochemical and anatomical substrates of depression and sickness behavior. Isr. J. Psychiatry Relat. Sci. 47, 64–71. 20686201

[B36] Jackson R. L. Alexander J. H. Maier S. F. (1980). Learned helplessness, inactivity, and associative deficits: effects of inescapable shock on response choice escape learning. J. Exp. Psychol. 6, 1. 10.1037/0097-7403.6.1.1 7373224

[B37] Jackson R. L. Maier S. F. Coon D. J. (1979). Long-term analgesic effects of inescapable shock and learned helplessness. Science 206, 91–93. 10.1126/science.573496 573496

[B38] Jackson R. L. Maier S. F. Rapaport P. M. (1978). Exposure to inescapable shock produces both activity and associative deficits in the rat. Learn. Motiv. 9, 69–98. 10.1016/0023-9690(78)90027-9

[B39] Jones M. E. Lebonville C. L. Barrus D. Lysle D. T. (2015). The role of brain interleukin-1 in stress-enhanced fear learning. Neuropsychopharmacology 40, 1289. 10.1038/npp.2014.317 25430780 PMC4367475

[B40] Jones M. E. Lebonville C. L. Paniccia J. E. Balentine M. E. Reissner K. J. Lysle D. T. (2018). Hippocampal interleukin-1 mediates stress-enhanced fear learning: a potential role for astrocyte-derived interleukin-1β. Brain Behav. Immun. 67, 355–363. 10.1016/j.bbi.2017.09.016 28963000 PMC5696098

[B41] Kessler R. C. Sonnega A. Bromet E. Hughes M. Nelson C. B. (1995). Posttraumatic stress disorder in the National Comorbidity Survey. Arch. Gen. Psychiatry 52, 1048–1060. 10.1001/archpsyc.1995.03950240066012 7492257

[B42] Kilpatrick D. G. Resnick H. S. Milanak M. E. Miller M. W. Keyes K. M. Friedman M. J. (2013). National estimates of exposure to traumatic events and PTSD prevalence using DSM-IV and DSM-5 criteria. J. Traumatic. Stress 26, 537–547. 10.1002/jts.21848 24151000 PMC4096796

[B43] Landgraf D. Long J. Der-Avakian A. Streets M. Welsh D. K. (2015). Dissociation of learned helplessness and fear conditioning in mice: a mouse model of depression. PLoS ONE 10:e0125892. 10.1371/journal.pone.0125892 25928892 PMC4416012

[B44] Liu X. Wu R. Tai F. Ma L. Wei B. Yang X.. (2013). Effects of group housing on stress induced emotional and neuroendocrine alterations. Brain Res. 1502, 71–80. 10.1016/j.brainres.2013.01.044 23380532

[B45] Long V. A. Fanselow M. S. (2012). Stress-enhanced fear learning in rats is resistant to the effects of immediate massed extinction. Stress 15, 627–636. 10.3109/10253890.2011.650251 22176467 PMC4000451

[B46] Maier S. F. (1984). Learned helplessness and animal models of depression. Progr. Neuropsychopharmacol. Biol. Psychiatry 8, 435–46. 10.1016/0278-5846(84)90120-9 6385140

[B47] Maier S. F. (1990). Role of fear in mediating shuttle escape learning deficit produced by inescapable shock. J. Exp. Psychol. Anim. Behav. Process. 16, 137–149. 10.1037//0097-7403.16.2.137 2335769

[B48] Maier S. F. (2001). Exposure to the stressor environment prevents the temporal dissipation of behavioral depression/learned helplessness. Biol. Psychiatry 49, 763–773. 10.1016/S0006-3223(00)01095-7 11331084

[B49] Maier S. F. Grahn R. E. Kalman B. A. Sutton L. C. Wiertelak E. P. Watkins L. R. (1993). The role of the amygdala and dorsal raphe nucleus in mediating the behavioral consequences of inescapable shock. Behav. Neurosci. 107:377. 10.1037/0735-7044.107.2.377 8484901

[B50] Maier S. F. Seligman M. E. (2016). Learned helplessness at fifty: insights from neuroscience. Psychol. Rev. 123, 349. 10.1037/rev0000033 27337390 PMC4920136

[B51] Maier S. F. Watkins L. R. (1995). Intracerebroventricular interleukin-1 receptor antagonist blocks the enhancement of fear conditioning and interference with escape produced by inescapable shock. Brain Res. 695, 279–282. 10.1016/0006-8993(95)00930-O 8556346

[B52] Metzger M. Bueno D. Lima L. B. (2017). The lateral habenula and the serotonergic system. Pharmacol. Biochem. Behav. 162, 22–28. 10.1016/j.pbb.2017.05.007 28528079

[B53] Meyer E. M. Long V. Fanselow M. S. Spigelman I. (2013). Stress increases voluntary alcohol intake, but does not alter established drinking habits in a rat model of posttraumatic stress disorder. Alcoholism 37, 566–574. 10.1111/acer.12012 23126586 PMC3567303

[B54] Minor T. Plumb T. Schell C. Pham A. (2010). Brain Adenosine Signaling in Psychological Trauma and Comorbid Depression. Neurobiology of Post-Traumatic Stress Disorder. New York, NY: Nova Science Publishers, Inc, 229–257.

[B55] Minor T. R. Chang W. C. Winslow J. L. (1994a). Stress and adenosine: I. Effect of methylxanthine and amphetamine stimulants on learned helplessness in rats. Behav Neurosci. 108, 254–264. 10.1037/0735-7044.108.2.254 8037869

[B56] Minor T. R. Dess N. K. Ben-David E. Chang W.-C. (1994c). Individual differences in vulnerability to inescapable shock in rats. J. Exp. Psychol. 20, 402. 10.1037/0097-7403.20.4.402 7964522

[B57] Minor T. R. Dess N. K. Overmier J. B. (1991). “Inverting the traditional view of “learned helplessness”,” in Fear, Avoidance, and Phobias: A Fundamental Analysis, ed Denny M. R. (Hillsdale, NJ: Lawrence Erlbaum Associates, Inc.), 87–133.

[B58] Minor T. R. Huang Q. Witt A. E. (2006). Cytokine-purine interactions in traumatic stress, behavioral depression, and sickness. CNS Neurol. Disord. Drug Targets 5, 547–560. 10.2174/187152706778559282 17073657

[B59] Minor T. R. Hunter A. M. (2002). Stressor controllability and learned helplessness research in the United States: sensitization and fatigue processes. Integr. Physiol. Behav. Sci. 37, 44–58. 10.1007/BF02688805 12069365

[B60] Minor T. R. Jackson R. L. Maier S. F. (1984). Effects of task-irrelevant cues and reinforcement delay on choice-escape learning following inescapable shock: evidence for a deficit in selective attention. J. Exp. Psychol. 10, 543. 10.1037//0097-7403.10.4.543 6491612

[B61] Minor T. R. LoLordo V. M. (1984). Escape deficits following inescapable shock: the role of contextual odor. J. Exp. Psychol. 10:168 10.1037//0097-7403.10.2.168

[B62] Minor T. R. Pelleymounter M. A. Maier S. F. (1988). Uncontrollable shock, forebrain norepinephrine, and stimulus selection during choice-escape learning. Psychobiology 16, 135–145.

[B63] Minor T. R. Plumb T. N. (2012). Learned Helplessness, Encyclopedia of the Sciences of Learning. Berlin: Springer, 1740–1743. 10.1007/978-1-4419-1428-6_277

[B64] Minor T. R. Rowe M. Cullen P. K. Furst S. (2008). Enhancing brain adenosine signaling with the nucleoside transport blocker NBTI (S-(4-nitrobenzyl)-6-theoinosine) mimics the effects of inescapable shock on later shuttle-escape performance in rats. Behav. Neurosci. 122, 1236–1247. 10.1037/a0013143 19045943

[B65] Minor T. R. Rowe M. K. Job R. S. Ferguson E. C. (2001). Escape deficits induced by inescapable shock and metabolic stress are reversed by adenosine receptor antagonists. Behav. Brain Res. 120, 203–212. 10.1016/S0166-4328(00)00376-4 11182168

[B66] Minor T. R. Saade S. (1997). Poststress glucose mitigates behavioral impairment in rats in the “learned helplessness” model of psychopathology. Biol. Psychiatry 42, 324–334. 10.1016/S0006-3223(96)00467-2 9276072

[B67] Minor T. R. Winslow J. L. Chang W. C. (1994b). Stress and adenosine: II. Adenosine analogs mimic the effect of inescapable shock on shuttle-escape performance in rats. Behav. Neurosci. 108, 265–276. 10.1037/0735-7044.108.2.265 8037870

[B68] Overmier J. B. Seligman M. E. (1967). Effects of inescapable shock upon subsequent escape and avoidance responding. J. Comp. Physiol. Psychol. 63, 28. 10.1037/h0024166 6029715

[B69] Pennington Z. T. Anderson A. S. Fanselow M. S. (2017). The ventromedial prefrontal cortex in a model of traumatic stress: fear inhibition or contextual processing? Learn Memory 24, 400–406. 10.1101/lm.046110.117 PMC5580532 28814465

[B70] Perusini J. N. Meyer E. M. Long V. A. Rau V. Nocera N. Avershal J.. (2016). Induction and expression of fear sensitization caused by acute traumatic stress. Neuropsychopharmacology 41, 45. 10.1038/npp.2015.224 26329286 PMC4677128

[B71] Plumb T. N. Cullen P. K. Minor T. R. (2015). Parameters of hormetic stress and resilience to trauma in rats. Stress 18, 88–95. 10.3109/10253890.2014.974154 25319800

[B72] Plumb T. N. Sterlace S. R. Cavanaugh K. A. Minor T. R. (2013). Stress, Brain Adenosine Signaling, and Fatigue-Related Behavioral Processes. Berlin: Adenosine; Springer, 535–558. 10.1007/978-1-4614-3903-5_25

[B73] Poulos A. M. Reger M. Mehta N. Zhuravka I. Sterlace S. S. Gannam C. (2014). Amnesia for early life stress does not preclude the adult development of posttraumatic stress disorder symptoms in rats. Biol. Psychiatry 76, 306–314. 10.1016/j.biopsych.2013.10.007 24231200 PMC3984614

[B74] Rau V. DeCola J. P. Fanselow M. S. (2005). Stress-induced enhancement of fear learning: an animal model of posttraumatic stress disorder. Neurosci. Biobehav. Rev. 29, 1207–1223. 10.1016/j.neubiorev.2005.04.010 16095698

[B75] Rau V. Fanselow M. S. (2009). Exposure to a stressor produces a long lasting enhancement of fear learning in rats. Stress 12, 25–133. 10.1080/10253890802137320 18609302

[B76] Richter-Levin G. Stork O. Schmidt M. V. (2018). Animal models of PTSD: a challenge to be met. Mol. Psychiatry. 10.1038/s41380-018-0272-5 30816289 PMC6756084

[B77] Rytwinski N. K. Scur M. D. Feeny N. C. Youngstrom E. A. (2013). The co-occurrence of major depressive disorder among individuals with posttraumatic stress disorder: a meta-analysis. J. Traumatic. Stress. 26, 299–309. 10.1002/jts.21814 23696449

[B78] Seligman M. E. Maier S. F. (1967). Failure to escape traumatic shock. J. Exp. Psychol. 74, 1. 10.1037/h0024514 6032570

[B79] Servatius R. J. Ottenweller J. E. Natelson B. H. (1995). Delayed startle sensitization distinguishes rats exposed to one or three stress sessions: further evidence toward an animal model of PTSD. Biol. Psychiatry 38, 539–546. 10.1016/0006-3223(94)00369-E 8562666

[B80] Shors T. J. (2004). Learning during stressful times. Learn. Memory 11, 137–144. 10.1101/lm.66604 15054128 PMC3364672

[B81] Short K. R. Maier S. F. (1993). Stressor controllability, social interaction, and benzodiazepine systems. Pharmacol. Biochem. Behav. 45, 827–835. 10.1016/0091-3057(93)90128-G 8415822

[B82] Steenbergen H. L. Heinsbroek R. P. Van Haaren F. Van de Poll N. E. (1989). Sex-dependent effects of inescapable shock administration on behavior and subsequent escape performance in rats. Physiol. Behav. 45, 781–787. 10.1016/0031-9384(89)90295-3 2780848

[B83] Strong P. V. Christianson J. P. Loughridge A. B. Amat J. Maier S. F. Fleshner M. (2011). Greenwood, B. N., 5-hydroxytryptamine 2C receptors in the dorsal striatum mediate stress-induced interference with negatively reinforced instrumental escape behavior. Neuroscience 197, 132–44. 10.1016/j.neuroscience.2011.09.041 21958863 PMC3235414

[B84] Szczytkowski-Thomson J. L. Lebonville C. L. Lysle D. T. (2013). Morphine prevents the development of stress-enhanced fear learning. Pharmacol. Biochem. Behav. 103, 672–677. 10.1016/j.pbb.2012.10.013 23159544

[B85] Tribble J. E. Fanselow M. S. (2019). Pair-housing rats does not protect from behavioral consequences of an acute traumatic experience. Behav. Neurosci. 133, 232–239. 10.1037/bne0000295 30628802 PMC6433510

[B86] Weiss J. M. (1968). Effects of coping responses on stress. J. Comp. Physiol. Psychol. 65, 251. 10.1037/h0025562 5668311

[B87] Weiss J. M. Goodman P. A. Losito B. G. Corrigan S. Charry J. M. Bailey W. H. (1981). Behavioral depression produced by an uncontrollable stressor: relationship to norepinephrine, dopamine, and serotonin levels in various regions of rat brain. Brain Res. Rev. 3, 167–205. 10.1016/0165-0173(81)90005-9

[B88] Will M. J. Watkins L. R. Maier S. F. (1998). Uncontrollable stress potentiates morphine's rewarding properties. Pharmacol. Biochem. Behav. 60, 655–664. 10.1016/S0091-3057(98)00027-6 9678649

[B89] Yehuda R. Antelman S. M. (1993). Criteria for rationally evaluating animal models of postraumatic stress disorder. Biol. Psychiatry 33, 479–486. 10.1016/0006-3223(93)90001-T 8513032

[B90] Zacharko R. M. Anisman H. (1991). Stressor-induced anhedonia in the mesocorticolimbic system. Neurosci. Biobehav. Rev. 15, 391–405. 10.1016/S0149-7634(05)80032-6 1956607

